# Cardiovascular magnetic resonance imaging in the prospective, population-based, Hamburg City Health cohort study: objectives and design

**DOI:** 10.1186/s12968-018-0490-7

**Published:** 2018-09-24

**Authors:** Sebastian Bohnen, Maxim Avanesov, Annika Jagodzinski, Renate B. Schnabel, Tanja Zeller, Mahir Karakas, Jan Schneider, Enver Tahir, Ersin Cavus, Clemens Spink, Ulf K. Radunski, Francisco Ojeda, Gerhard Adam, Stefan Blankenberg, Gunnar K. Lund, Kai Muellerleile

**Affiliations:** 1 University Heart Center Hamburg, Department of General and Interventional Cardiology, University Medical Center Hamburg-Eppendorf, Martinistrasse 52, 20246 Hamburg, Germany; 2 Department of Diagnostic and Interventional Radiology and Nuclear Medicine, University Medical Center Hamburg-Eppendorf, Martinistrasse 52, 20246 Hamburg, Germany; 3 Deutsches Zentrum für Herz-Kreislauf-Forschung e. V. (German Center for Cardiovascular Research), Partner Site Hamburg/Kiel/Lübeck, Germany, Hamburg, Germany

**Keywords:** Cardiovascular magnetic resonance, CMR, Population-based study, Coronary artery disease, Atrial fibrillation, Heart failure, T1 mapping, T2 mapping

## Abstract

**Background:**

The purpose of this work is to describe the objectives and design of cardiovascular magnetic resonance (CMR) imaging in the single center, prospective, population-based Hamburg City Health study (HCHS). The HCHS aims at improving risk stratification for coronary artery disease (CAD), atrial fibrillation (AF) and heart failure (HF).

**Methods:**

The HCHS will finally include 45,000 inhabitants of the city of Hamburg (Germany) between 45 and 74 years who undergo an extensive cardiovascular evaluation and collection of biomaterials. Risk-scores for CAD, AF and HF are used to create enriched subpopulations who are invited for CMR. A total number of approximately 12,362 subjects will undergo CMR and incident CAD, AF and HF will be assessed after 6 years follow-up. The standard CMR protocol includes cine-CMR, T1 and T2 mapping, aortic/mitral valve flow measurements, Late gadolinium enhancement, angiographies and measurements of aortic distensibility. A stress-perfusion scan is added in individuals at risk for CAD. The workflow of CMR data acquisition and analyses was evaluated in a pilot cohort of 200 unselected subjects.

**Results:**

The obtained CMR findings in the pilot cohort agree with current reference values and demonstrate the ability of the established workflow to accomplish the objectives of HCHS.

**Conclusions:**

CMR in HCHS promises novel insights into major cardiovascular diseases, their subclinical precursors and the prognostic value of novel imaging biomarkers. The HCHS database will facilitate combined analyses of imaging, clinical and molecular data (“Radiomics”).

## Background

### The Hamburg City health study

The single center, prospective, population-based cohort Hamburg City Health study (HCHS, www.hchs.hamburg) aims at identifying novel risk factors for major diseases such as coronary artery disease (CAD), atrial fibrillation (AF), heart failure (HF), dementia and stroke. HCHS will finally include 45,000 individuals between 45 and 74 years who undergo an extensive baseline evaluation of the cardiovascular and neurologic system. A detailed description of the overall study design and work flow will be published separately. One major focus of HCHS is the incremental prognostic value of cardiovascular magnetic resonance (CMR), which is performed in HCHS subpopulations at increased risk for incident CAD, AF and HF. This work describes the rationale and design of performing CMR in HCHS and reports the findings of a pilot-study.

### CMR in population based-studies

The armamentarium of CMR ranges from cine-CMR measurements of cardiac volumes, mass and function, over myocardial perfusion measurements, late gadolinium enhancement (LGE) CMR, native and post contrast T1- and T2 mapping CMR for assessing focal and diffuse myocardial injury to blood flow measurements by velocity-encoded (VENC) CMR. Cine-CMR is currently the reference method for quantification of cardiac volumes, mass and function [1]. Stress perfusion CMR provides a superior diagnostic accuracy for relevant stenosis of coronary arteries compared with radionuclide perfusion imaging [2, 3]. Furthermore, LGE serves as the semi-quantitative reference technique for depicting focal myocardial scar [4], whereas T1 and T2 mapping techniques offer a quantitative assessment of diffuse myocardial injury [5]. Most importantly, proof of myocardial fibrosis by LGE CMR was consistently found to be associated with adverse outcomes in ischemic and non-ischemic cardiomyopathies [6, 7]. Furthermore, arterial stiffness is an independent predictor for the development of cardiovascular disease and cardiovascular mortality [8] and can be assessed non-invasively by aortic distensibility (AD) and the pulse wave velocity (PWV) on CMR [8]. In addition, VENC CMR enables accurate blood flow measurements for a quantitative assessment of valvular heart disease [9]. A major general strength of CMR for research application is an excellent reproducibility of quantitative measurements compared with other techniques, such as echocardiography [10, 11]. CMR is therefore an attractive instrument for population-based studies [12] and is implemented in several ongoing population-based studies, such as the UK Biobank [13], the German National Cohort [14] and the Canadian Alliance for Healthy Hearts and Minds cohort study (CAHHM) [15].

### Coronary artery disease

Non-invasive stress testing is the key diagnostic tool in *symptomatic* suspected or known, stable CAD [16, 17], but there are only limited data supporting stress testing for CAD risk-assessment in *asymptomatic* individuals [18–20]. Consequently, stress testing is currently not recommended in asymptomatic individuals without known CAD [20, 21]. However, CMR has great potential in this context, since stress-perfusion CMR offers a superior diagnostic accuracy to depict myocardial ischemia compared to other non-invasive stress tests [2, 3] and provides important prognostic information [22, 23]. Moreover, LGE CMR is the non-invasive reference technique to detect occult myocardial scar, which is a strong predictor of major adverse events and mortality [24, 25]. Thus, HCHS promises unique data on the value of stress CMR in individuals at risk for CAD.

### Atrial fibrillation

Recent studies indicate that cardiac imaging could improve the prediction of AF [26, 27]. In particular, CMR is currently used as the reference standard for measurements of left atrial (LA) volumes and function [28], which were recently found to be associated with stroke risk [29]. HCHS evaluates the potential incremental value of combining well-established risk factors, novel biomarkers and CMR measures of LA and left ventricular (LV) volumes, mass and -function, as well as myocardial tissue composition to identify patients at risk for incident AF.

### Heart failure

There is a strong need to depict individuals at risk for HF before clinical manifestation to initiate prevention and therapy as early as possible in advance of clinical events. LV-ejection fraction (EF) is traditionally the most important functional cardiac parameter in HF [30, 31]. However, the value of LVEF is limited by definition in HF with preserved EF (HFpEF) [31]. In HFpEF patients, LA volume and LV mass were independent predictors of morbidity and mortality [32], but CMR offers several tools beyond conventional imaging in this context. In particular, tissue characterisation by T1 mapping CMR with quantification of native T1 and extracellular volume (ECV) is extremely promising, due to its ability to quantify diffuse myocardial fibrosis, which is typically undetected by LGE [33, 34] and myocardial fibrosis is associated with an unfavourable prognosis in several conditions [6, 7]. HCHS evaluates the potential incremental value of combining CMR tissue characterization with circulating biomarkers to yield an optimized HF prediction.

## Methods

### General

The local ethics committee approved the HCHS. All participants are informed explicitly on direct (such as contrast media), but also indirect risks (such as downstream testing) of participating in HCHS. Written informed consent is obtained in all subjects of the pilot phase and of the main study. HCHS will finally include 45,000 participants between 45 and 74 years of age within a period of 6 years for baseline examination. The final HCHS-population will contain 50% males and 50% females. Briefly, the recruitment pathway will be as follows: Firstly, the Residents’ Registration office of the city of Hamburg will draw a sample of 20,000 citizens from the central population register every second year. The only pre-selection criteria will be age between 45 and 74 years and gender. Secondly, the Residents’ Registration office will send the contact data of these 20,000 potential HCHS participants to the HCHS recruitment center. Thirdly, the HCHS recruitment center will then subsequently invite these individuals without further stratification or knowledge of clinical characteristics to participate in the HCHS. Individuals, who agree to participate in the HCHS are then scheduled for baseline evaluation. All participants will be invited to at least one follow-up visit, 6 years after the baseline visit. Baseline and follow-up visits are performed in the HCHS study center, which is located in a dedicated building at the University Medical Center Hamburg-Eppendorf, Hamburg, Germany. The participants undergo interviews and questionnaires, including a comprehensive assessment of psychosocial, environmental and lifestyle risk factors such as nutrition, physical activity or professional life. All participants undergo spirometry, blood-pressure measurements, an electrocardiogram, an ultrasound of the carotid arteries, 2D- and 3D echocardiography and cognitive testing. Furthermore, the following biomaterials are collected, processed and stored within HCHS: serum, plasma, blood cells (erythrocytes, peripheral mononuclear cells (PBMCs), nucleic acids, including genomic DNA (extracted from leucocytes) and RNA (extracted from PBMCs and whole blood), urine, saliva, tonsil swab, liquid from the interdental spaces, skin stanza for production of induced pluripotent stem cells. Prospectively, the following assays/measurements will be used: a) Immunological and clinical-chemistry assays for protein-based measurements and b) whole genome sequencing and targeted SNP genotyping using TaqMan assays. Further measurements will be used on a project-specific basis.

Data from the baseline visit are used to calculate conventional risk-scores for the HCHS target diseases: Individuals at risk for CAD are identified by the ESC Euro SCORE (Systematic COronary Risk Evaluation) > 4%, which has the advantage that a modified version for the German population exists [35, 36]. The CHARGE-AF score is used as the first and best validated risk prediction algorithm for incident atrial fibrillation in the community to identify participants at intermediate or high risk (> 6%) for AF [37]. Individuals at increased risk for HF (> 3.4%) are identified by the optimized ARIC-Heart-Failure-Prediction-Score, which includes n-terminal pro b-type natriuretic peptide (NT-proBNP) [38]. Participants at increased risk for CAD, AF and/or HF are then invited for CMR in order to create enriched CMR subpopulations.

Specific contraindications for CMR within HCHS are defined as claustrophobia, possible pregnancy, any tattoo on the upper side of the body, implanted cardioverter defibrillators or pacemakers, cochlear implants, insulin pumps or similar devices, intrauterine spirals and any other implanted ferromagnetic material. Furthermore, each participant is informed explicitly and in detail about recent findings on deposition of gadolinium in the brain after administration of gadolinium-based contrast agents [39]. Although, gadoterate meglumine (Dotarem®, Guerbet, Aulnay, France) seems to be safe in this context in humans [39], the participants have the opportunity for a conscious and informed decision on this topic. Any reduction in estimated glomerular filtration rate (eGFR) below 60 mL/min is regarded as a contraindication for contrast enhanced CMR within HCHS.

### Study population

**Fig. 1:**
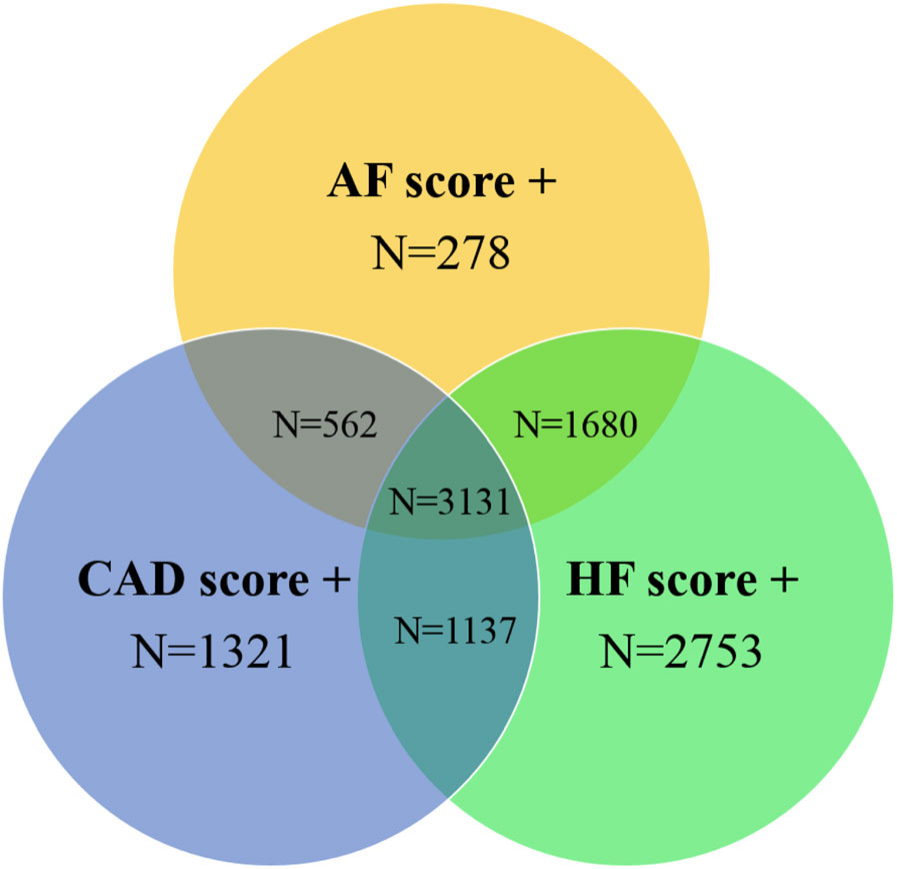
Expected overlap between participants with a positive risk score for CAD, AF and HF. Abbreviations: *CAD = Coronary artery disease, AF = atrial fibrillation, HF = heart failure*

In HCHS, we expect 10,252 individuals with an ESC Euro Score > 4%; 9418 with a risk score for AF > 6% and 14,502 with HF risk score > 3.4%. Assuming that 60% of the participants accept invitation for CMR, which was deduced from the German National Cohort [14], we expect to perform CMR in 6151; 5651 and 8701 individuals at increased risk for CAD, AF and HF, respectively. Recruitment rates will be continuously tracked and if the recruitment rate is lower than expected, the HCHS steering committee will decide on a potential additional recruitment phase. Figure 1 illustrates the expected overlap between the three groups. Furthermore, a cohort of 1500 unselected subjects serves as a reference population. The CMR subpopulation of HCHS will finally include approximately 12,362 subjects.

### CMR protocol

**Fig. 2:**
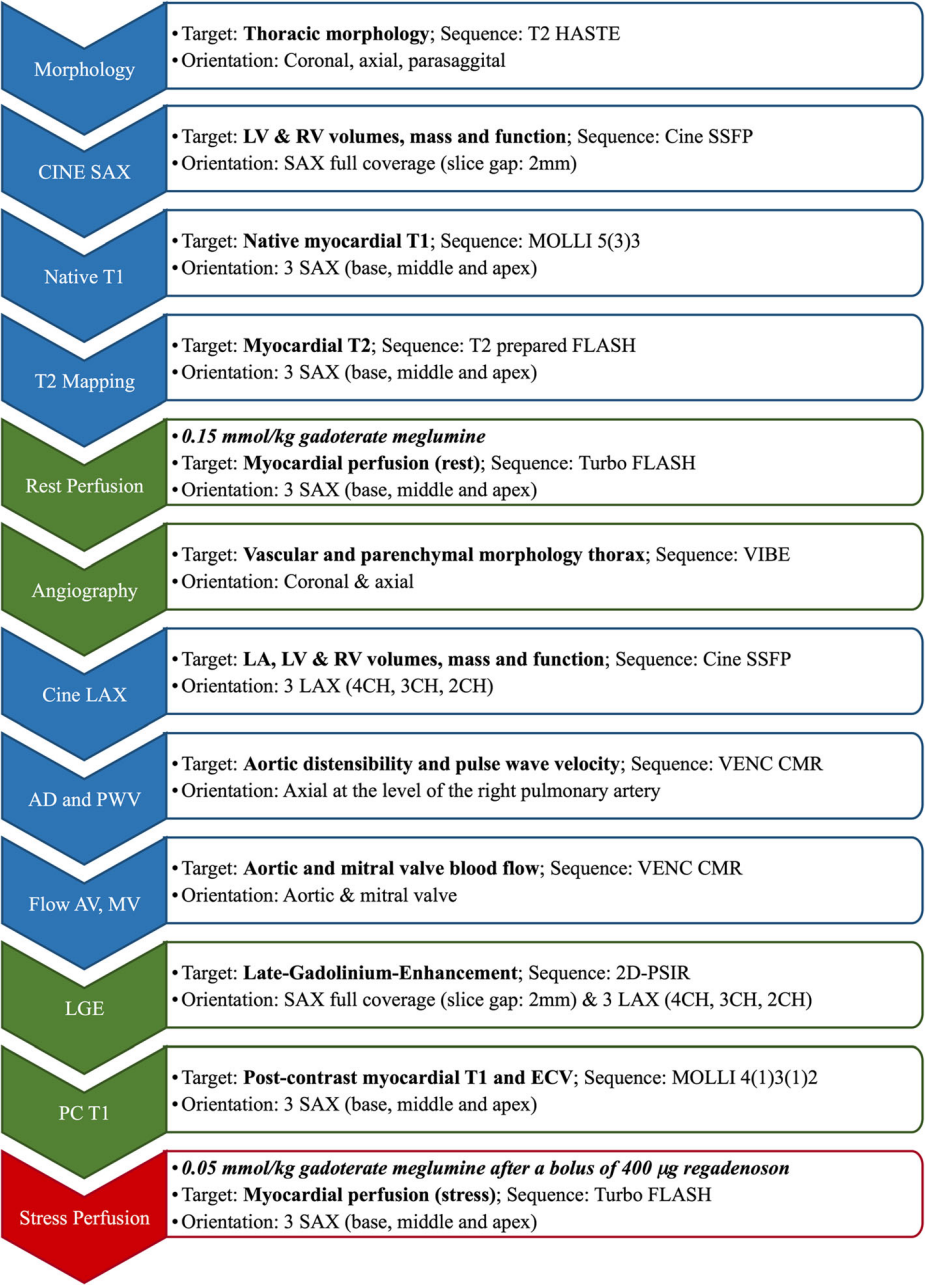
CMR protocol. Blue sequences indicate native CMR, green sequences indicate the use of contrast –media and red indicates stress perfusion. Abbreviations: *FLASH = fast low angle shot, HASTE = half fourier-acquired single shot turbo spin Echo, LAX = long axis view, LGE = late gadolinium enhancement, MOLLI = modified Look-Locker inversion recovery, PC = post-contrast, PSIR = phase sensitive inversion recovery, SAX = short axis view, bSSFP = balanced steady state free precession, VENC = velocity encoded, VIBE = volumetric interpolated breath-hold examination, 4CH = 4 chamber view, 3CH = 3 chamber view, 2CH = 2 chamber view*

CMR is exclusively performed on a dedicated 3 T scanner (MAGNETOM™ Skyra, Siemens Healthineers, Erlangen, Germany), which is located at the University Medical Center Hamburg-Eppendorf, Hamburg, Germany. Three different CMR protocols are implemented in HCHS (Fig. 2):

- First, the standard protocol includes balanced steady state free-precession (bSSFP) cine-CMR, native and post-contrast T1 mapping (5b(3b)3b and 4b(1b)3b(1b)2b *modified Look-Locker inversion recovery* (MOLLI) schemes, respectively), T2 mapping (T2 prepared, fast-low-angle shot (FLASH) sequence), rest perfusion (Turbo FLASH sequence) during the first-pass of 0.15 mmol/kg gadoterate meglumine (Dotarem®, Guerbet, Aulnay, France), an angiography of the thoracic vessels (volumetric interpolated breath-hold examination, volumetric interpolated breath-hold examination (VIBE) sequence), 2D-cine and flow measurements of the ascending and descending aorta at the level of the right pulmonary artery for calculation of AD and PWV, 2D aortic and mitral valve flow measurements as well as LGE imaging (phase sensitive inversion recovery, PSIR).
- Second, a reduced non-contrast protocol is performed in subjects who decline the use of gadoterate meglumine (Fig. 2).
- Third, the stress protocol consists of the standard protocol followed by a stress perfusion scan (Turbo FLASH sequence) during an additional first-pass of 0.05 mmol/kg gadoterate meglumine 1 min after the administration of 400 μg regadenoson (Rapiscan, Pharma Solutions, London, United Kingdom).

The CMR protocols and typical imaging parameters are presented in Table 1 and Fig. 2.

**Table 1:** CMR sequence parameters

Modality	Pulse sequence	Voxel size (mm^3^)	FoV (mm^2^)	TR (ms)	TE (ms)	FA (°)	PAT
Morphology	T2 HASTE	1.3 × 1.3 × 8	400	1100	90	110	2
Cine CMR	bSSFP	1.6 × 1.6 × 8	340	48	1.5	80	3
Native T1 Mapping	MOLLI 5b(3b)3b	1.4 × 1.4 × 8	360	281	1.1	35	2
T2 Mapping	T2 prepared FLASH	1.9 × 1.9 × 8	360	207	1.3	12	2
Perfusion CMR	Turbo FLASH	1.9 × 1.9 × 8	360	158	1	10	2
Angiography	VIBE	1.4 × 1.4 × 5	380	3.3	1.3	9	4
Flow measurements	VENC	1.8 × 1.8 × 6	340	46	2.5	20	2
AD and PWV	VENC	1.2 × 1.2 × 8	300	17	2.3	20	2
LGE	2D-PSIR	1.6 × 1.6 × 8	400	700	1.2	55	2
PC T1 Mapping	MOLLI 4b(1b)3b(1b)2b	1.4 × 1.4 × 8	360	361	1.1	35	2

*Abbreviations: AD* Aortic Distensibility, *FA* flip angle, *FLASH* fast low angle shot, *FoV* reconstructed field of view, *HASTE* Half fourier-acquired single shot turbo spin Echo, *LAX* long axis view, *LGE* late gadolinium enhancement, *MOLLI* modified Look-Locker inversion recovery, *PAT* parallel acquisition technique, *PC* post-contrast, *PSIR* phase sensitive inversion recovery, *PWV* pulse wave velocity, *SAX* short axis view, *bSSFP* balanced steady state free precession, *TE* echo time, *TR* repetition time, *VENC* velocity encoded, *VIBE* volumetric interpolated breath-hold examination

### CMR data analysis

A standardized workflow for qualitative and quantitative image analysis was implemented in order to provide accurate and reproducible data but also to ensure a standardized handling of incidental findings. In particular, standard operating procedures (SOPs) were established for all qualitative and quantitative items in agreement with current Society for Cardiovascular Magnetic Resonance (SCMR) recommendations [40]. A second, blinded observer analyzes every fifths study of the main study order to assess inter-observer variability and to maintain stable data quality. Each CMR data set is analyzed on the day of acquisition by a radiologist or cardiologist with at least 2 years of CMR experience under supervision by a SCMR/European Association of Cardiovascular Imaging (EACVI) level III approved radiologist or cardiologist. All CMR data analyses will be performed using the commercially available and established software cvi42 (Circle Cardiovascular Imaging Inc., Calgary, Alberta, Canada).

**Fig. 3:**
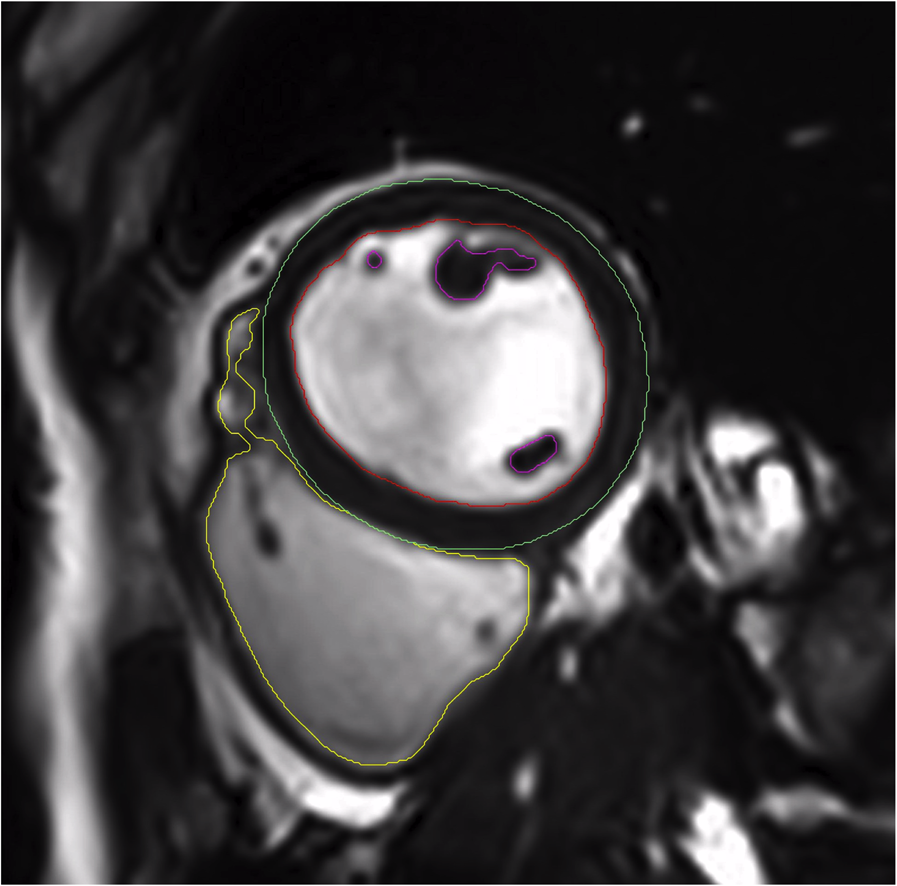
Cine CMR analysis. A typical mid-ventricular end-diastolic short-axis slice is shown with left-ventricular endocardial (red), papillary muscle (purple) and epicardial (green) contours as well as right-ventricular endocardial contour (yellow)

**Fig. 4:**
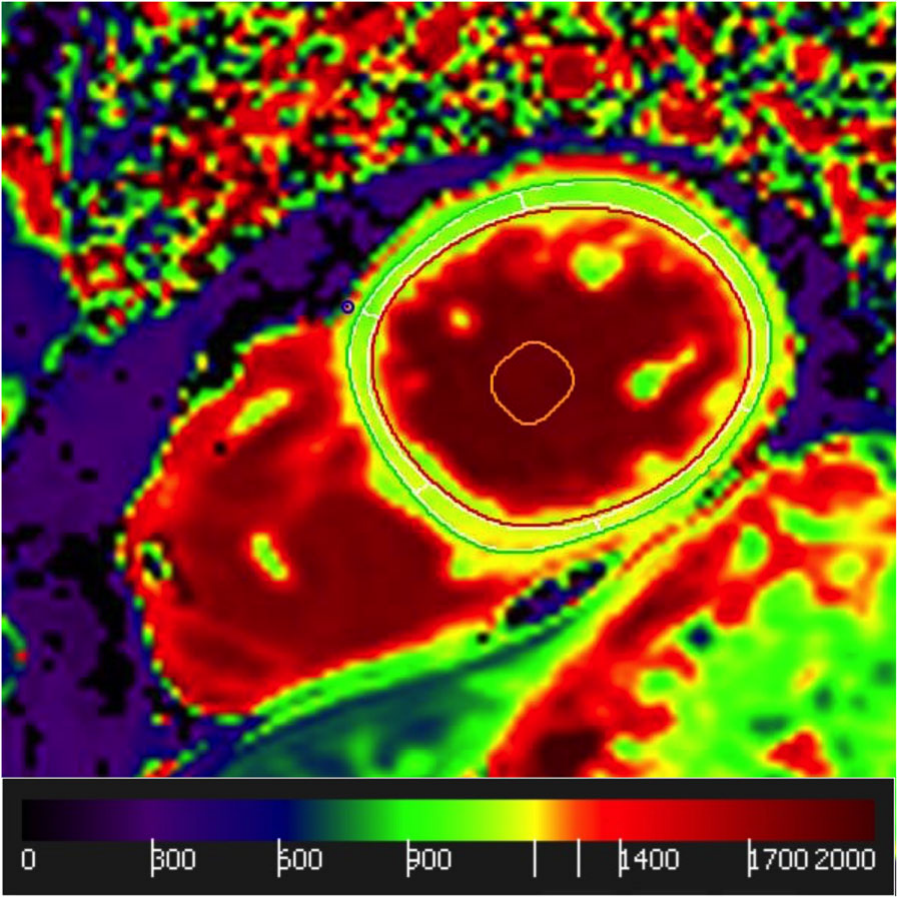
T1 mapping analysis. Endo- (red) and epicardial (green) contours of a representative native T1 map. A 10% endo- and epicardial offset was applied to avoid contamination by blood pool or epicardial tissue. The yellow contour represents the blood pool measurement for ECV calculation

**Fig. 5:**
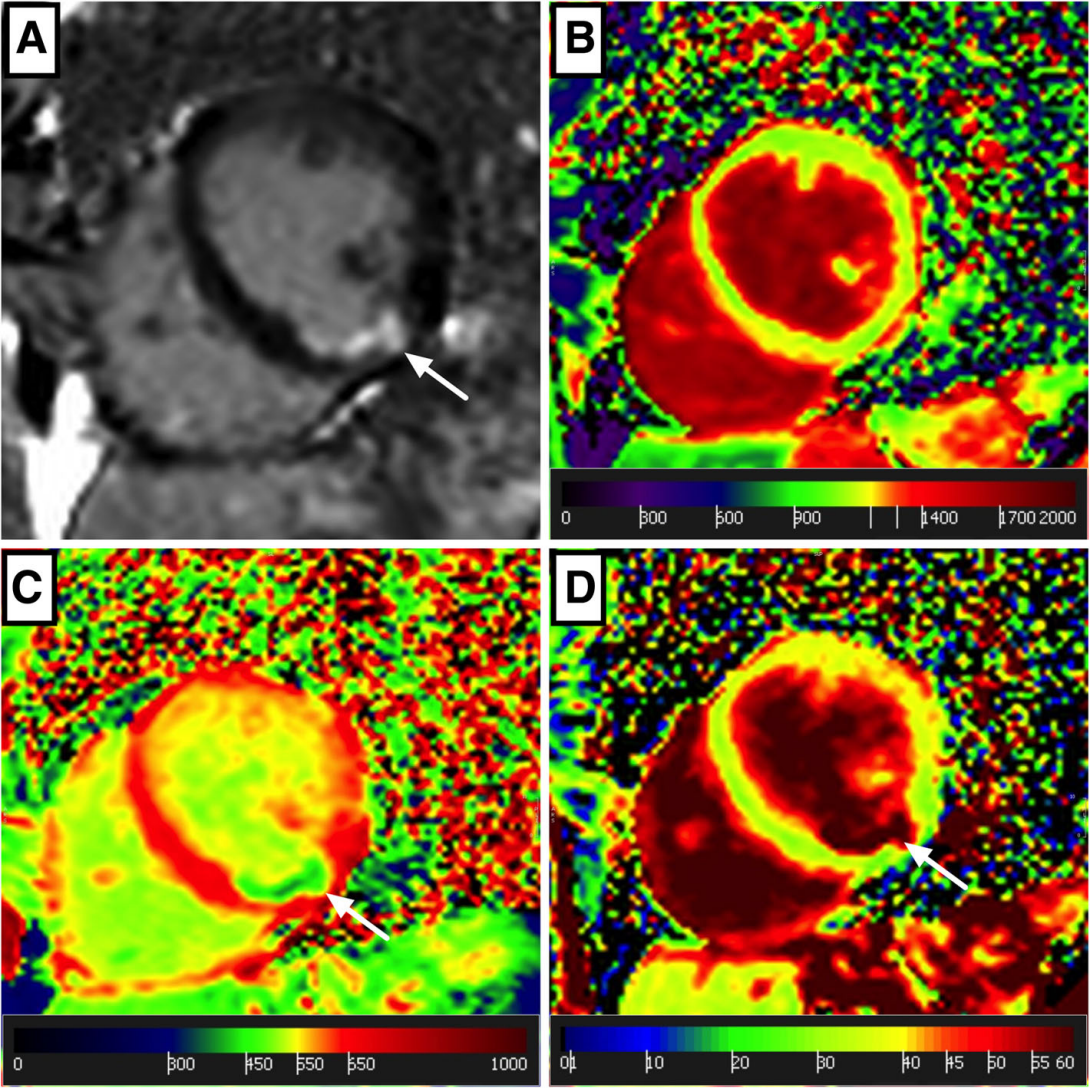
Example of a clinically “silent” myocardial infarction. Native mapping and contrast enhanced CMR from a subject with no history of cardiac disease. Arrows indicate a typically ischemic, subendocardial scar on the late gadolinium enhancement (LGE) image (**a**) in the inferior wall. There was no regional increase in native myocardial T1 (**b**), but a decrease in post-contrast T1 (**c**) with subsequent increase in extracellular volume fraction (ECV) (**d**). The constellation of an ischemic scar without increased native T1 or T2 values was interpreted as a previous, clinically “silent” myocardial infarction in heretofore-unknown coronary artery disease

The detailed workflow for CMR data analysis is as follows:

1. Endocardial LV and right-ventricular (RV) and epicardial LV contours are manually traced on end-diastolic and end-systolic short axis cine-CMR images from base to apex including the papillary muscles for calculating LV and RV volumes, mass and function as recommended [40] (Fig. 3). LA and right atrial (RA) volumes are calculated using the biplane long-axis method. In addition feature tracking for myocardial strain analysis is performed on cine-CMR images.
2. Myocardial T2 values are obtained on three representative short axes (base, middle and apex) as recently established [41].
3. Native and post-contrast T1 as well as ECV are obtained from corresponding short axis maps by carefully drawing endo- and epicardial contours with 10% endo- and epicardial offsets to avoid contamination by blood pool or epicardial tissue as recommended [5] (Fig. 4).
4. Myocardial perfusion at rest and at stress are qualitatively assessed as recommended [40].
5. LGE images are evaluated qualitatively (Fig. 5) and by using the semi-quantitative 5-SD threshold technique in ischemic LGE and the 3-SD threshold technique in non-ischemic LGE as recommended [40].
6. Through-plane VENC flow measurements are analyzed for the aortic and mitral valve by placing regions of interest (ROI) at the tip of the aortic valve cusps and mitral valve leaflets, respectively. An additional ROI is placed into stationary tissue to correct for phase-offsets [40].
7. Aortic dimensions are obtained from the angiography at the level of the aortic sinus, at the sino-tubular junction as well as at the ascending and descending thoracic aorta [42].
8. Aortic distensibility (AD) is assessed from a transverse 2D through plane phase-contrast cine CMR of the ascending and descending aorta at the level of the right pulmonary artery according to the formula: AD (1/mmHg*10^3^) = (A_max_ – A_min_) / A_min_ x (P_max_ – P_min_).

*A*_*max*_ *and A*_*min*_ *represent the maximal and minimal cross-sectional area on cine CMR images of the aorta at the level of the right pulmonary artery and P*_*max*_ *and P*_*min*_ *represent the systolic and diastolic blood pressure* [43]. PWV in the aortic arch is estimated from the temporal difference between two blood flow wave fronts according to the formula: PWV (m/s) = ∆x / ∆t.

*∆x is the distance between the ascending and descending aorta at the level of the right pulmonary artery and ∆t is the time difference between the arrival of 2 wavefronts, using the transit-time method from the corresponding velocity-time curves of the ascending and descending aorta* [44].

### Study database

All quantitative data are automatically transferred from cvi42 into a dedicated study database. Data from quantitative tissue characterization by T1 and T2 mapping as well as LGE are exported as mean global values, but also for 16 myocardial segments according to the American Heart Association (AHA) model [45]. Qualitative data are documented in highly standardized forms and transferred automatically into the study database.

### Extra-cardiac incidental findings

The acquired data volume is systematically screened for extra-cardiac incidental findings. SOPs are established for all organs in the scanning volume with findings categorized into three groups as “no relevant finding”, such as singular uncomplicated renal cysts, “relevant finding requiring elective medical assessment”, such as a suspicious liver lesion or “relevant finding requiring immediate medical assessment”, such as a pulmonary artery embolism. Clinical experts for the involved organs/entities discuss all ambiguous findings in a weekly conference before reporting relevant findings requiring elective medical assessment. Participants with relevant findings requiring immediate medical assessment are advised to present immediately at the next emergency department.

### Reporting of CMR findings

A standardized CMR report is automatically generated which is suitable for presentation to the general practitioner. In order to facilitate handling of CMR findings for general practitioners, a simple three-step system was implemented with a concise categorization of findings into “normal”, “abnormal requiring elective medical assessment” or “abnormal requiring immediate medical assessment”.

### Statistical analysis

Time-to-event methods will be used for identifying and assessing risk factors for mortality and incidence or progression of diseases. For example, Cox proportional hazards regression and its extensions will be used to simultaneously investigate the effects of risk factors of interest while adjusting for potential confounders. We will employ alternative approaches like Poisson regression, which allows the incorporation of baseline mortality/incidence in the total Hamburg population or machine learning approaches like survival random forests. To quantify the improvement in risk prediction of a novel imaging marker or group of markers over endpoint-specific established risk factors, we will use among others the C-index and reclassification analyses and will assess the calibration of the models considered. The selection of the variables to be included in a model will be based on subject matter knowledge when possible. Newly developed risk algorithms will be tested in external cohorts to assess their generalizability. For repeated measurements, regression models for longitudinal data will be employed, such as generalized estimating equations, random effect models, and joint models for longitudinal and time-to-event data.

## Results

A pilot study was conducted to establish a robust and highly standardized CMR workflow, protocols and data analyses. In this pilot study, 200 unselected subjects underwent CMR. Sufficient image quality for obtaining major CMR parameters was achieved in 198 (99.0%) of the pilot participants. One-hundred-sixty-five (82.5%) participants accepted contrast-media application. Eight (4.8%) of these 165 participants had myocardial scar on LGE, including 6 participants with ischemic and 2 participants with non-ischemic patterns. ECV was available in 81 individuals in whom haematocrit values were obtained on the same day. Median ECV was 27 (IQR 26–29) % in these participants. Other major CMR findings in the pilot study are presented in Table 2. Mean inter-observer differences were 1.6 ± 5.3 mL/m^2^ for LVEDVi, 0.7 ± 7.8 g/m^2^ for LVMi, 1.1 ± 5.0% for LVEF, 2.8 ± 9.1 mL/m^2^ for RVEDVi, 0.4 ± 7.7% for RVEF, 0.6 ± 2.3 mL/m^2^ for LAVi, 0.4 ± 0.9 ms for myocardial T2, 4.7 ± 9.8 ms for native myocardial T1, respectively.

**Table 2:** Major CMR findings in the pilot-study cohort

Parameter (unit)	Values
Age (years)	63 (52–69)
Male sex (n, %)	101 (51)
BMI	25 (23–27)
Heart rate (bpm)	65 (60–74)
LVEDVi (mL/m^2^)	66 (57–77)
LVESVi (mL/m^2^)	19 (15–24)
LVSVi (mL/m^2^)	46 (39–52)
LVMi (g/m^2^)	64 (55–73)
LVEF (%)	71 (66–75)
RVEDVi (mL/m^2^)	71 (63–86)
RVESVi (mL/m^2^)	29 (22–35)
RVSVi (mL/m2)	45 (36–52)
RVEF (%)	60 (55–66)
LAVi (mL/m^2^)	29 (23–36)
Myocardial T2 (ms)	40 (39–42)
Native myocardial T1 (ms)	1179 (1153–1207)

*Abbreviations: BMI* body-mass-index, *LV* left ventricular, *RV* right ventricular, *EDVi* end-diastolic volume index, *ESVi* end-systolic volume index, *SVi* stroke volume index, *Mi* mass index, *EF* ejection fraction, *LA* left atrial, Numbers are median (interquartile range) for continuous and n (% of total column number) for categorical data

## Discussion

The aim of CMR imaging in the HCHS is to improve risk stratification for the major cardiac diseases of CAD, AF and HF by establishing imaging biomarkers as correlates for subclinical alterations of myocardial function and/or tissue composition. In particular, the HCHS database will facilitate combined analyses of imaging, clinical and molecular data (“Radiomics”), which may ultimately include advanced “machine-learning” algorithms. The use of pre-selected, enriched populations for the three target diseases constitutes one major difference compared to other ongoing population-based cohorts such as German National Cohort [14] and UK Biobank [13]. The potential advantage of this deviation from traditional, unselected population-based studies is to facilitate a better translation of the generated HCHS risk-scores into clinical reality. It can be expected that pre-selection will result in a higher number of subclinical abnormalities detected by CMR at baseline, but also in a higher incidence of target diseases during follow-up. Ultimately, HCHS is therefore not a conventional, purely descriptive population study, but also shares some characteristics with clinical studies. Although there will be no intervention, we expect that this study design will facilitate translation of our findings into clinical routine. For example, a potential future application could be to use CMR for identifying individuals with subclinical disease (e.g. with myocardial fibrosis) among individuals at risk, who may benefit from upstream therapeutic interventions. Furthermore, this study is unique in terms of applying some clinical CMR tools in a population-based context: In particular, the application of stress CMR for assessing myocardial ischemia constitutes a novelty in a population-based setting. We expect that the evaluation of stress-CMR within HCHS clarify the currently undefined role of stress testing in the large group of asymptomatic individuals who are at increased CAD risk [18–20]. A further important difference compared to the majority of recent population-based studies is the use of current quantitative tools for tissue characterization, namely T1 and T2 mapping, as well as the administration of contrast-media, which enables the detection and characterization of subtle, occult myocardial injury by LGE and ECV imaging. This aspect appears to be of particular importance, since unrecognized focal myocardial scar of ischemic or non-ischemic origin on LGE images [6, 24] or diffuse myocardial fibrosis by ECV imaging [7] are of crucial prognostic value in a variety of scenarios. We therefore hypothesize that the detection of subclinical, unrecognized myocardial pathologies by CMR significantly improve the risk stratification for CAD, AF and HF.

In the pilot study, we have established a highly standardized workflow for CMR data acquisition, analyses and handling to maintain stable and high-quality data throughout the HCHS. The findings in our pilot study match well with current reference values in the literature: The inter-observer differences were low and similar to recent studies [10, 11, 41]. Quantitative LV, RV and LA measures [42] as well as native myocardial T1 and T2 values match well with recently reported findings at 3 T [41, 42]. However, reproducibility could be different between healthy subjects and patients with cardiovascular disease. CMR participants of the pilot study where not preselected by the mentioned risk scores and could therefore be healthier compared to the final study population. Interestingly, we found myocardial scar by LGE imaging in 4.8% of pilot subjects. This rate is a little lower than we expect to find in the main study, since our pilot study included unselected participants without increased risk for CAD or HF. Turkbey et al. [24] reported clinically “silent” myocardial scar in 7.9% of the participants of a significantly older 10-year follow-up population of the Multi Ethnic Study of Atherosclerosis (MESA) study. Nevertheless, our findings in unselected volunteers clearly highlight the unique ability of CMR to reveal occult myocardial disease as a potential powerful predictor of prognosis [24].

There are some potential limitations related to the study design of HCHS. Firstly, there is no selection/exclusion of ethnic groups, but participants have to be able to speak and understand German language, which inherently introduces some preselection. Secondly, the general HCHS population will consist of 50% men and 50% women, but gender will not be controlled in risk score positive subpopulations, since this would distort the scores and reduce the rates of incident target diseases at follow-up. Thus, male gender will inevitably be more represented in our CMR subpopulations. However, the unselected control group of 1500 individuals, who undergo CMR independent from risk-scoring, will compensate partially for this bias. Thirdly, the HCHS population could be not fully representative for other parts of Germany and Europe. However, the city of Hamburg includes urban, suburban and rural areas, so we believe that the results of HCHS can be translated to many other metropolitan regions. Nevertheless, transferability is certainly a limitation of all single site studies. Finally, some CMR parameters are notoriously site- and setting-specific, such as myocardial T1 and T2 values. Generalizability of findings in HCHS could therefore be limited, since HCHS is a single-center study with a single CMR 3 T scanner. We expect comparable ranges in settings with the same field strengths and vendor, such as in the German National Cohort [14], but findings in HCHS could be non-transferable to settings with 1.5 Tesla scanners, such as the UK Biobank [13]. One noteworthy feature of the HCHS CMR protocol is the performance of stress perfusion at the end of the scan after rest perfusion and LGE imaging (“rest-first” protocol). This deviation constitutes a compromise, which will allow us to keep a homogeneous core data set in the entire CMR population, but also to perform stress perfusion imaging in participants at risk for CAD in the same scan. The reason for applying a “rest-first” protocol is that ECV imaging cannot be performed under vasodilatation stress, since regadenoson-mediated vasodilation artificially affects the estimated ECV [46]. Otherwise, it would have been necessary to antagonize regadenoson, which is not feasible in a population study setting. Thus, the performance of a conventional “stress-first” protocol would mean waiving myocardial ECV, which is one of the most promising CMR parameters in a population context. We cannot exclude that the performance of stress perfusion CMR to depict myocardial ischemia could potentially be affected by the presence of a larger amount of contrast media after LGE imaging. However, “rest-first” protocols have been validated extensively earlier [47–49] and are currently accepted if performed with a sufficient interval between rest and stress perfusion [17, 50].

## Conclusions

CMR in HCHS promises novel insights into major cardiac diseases, their subclinical precursors and the prognostic value of novel imaging biomarkers. The HCHS database facilitate combined analyses of imaging, clinical and molecular data (“Radiomics”).

## Abbreviations

AD: Aortic distensibility
AF: Atrial fibrillation
AHA: American Heart Association
bSSFP: Balanced steady state free precession
CAD: Coronary artery disease
CMR: Cardiovascular magnetic resonance
ECV: Extracellular volume
EF: Ejection fraction
eGFR: Estimated glomerular filtration rate
FLASH: Fast-low-angle shot
HCHS: Hamburg City Health study
HF: Heart failure
HFpEF: Heart failure with preserved ejection fraction
LA: Left atrium/left atrial
LGE: Late gadolinium enhancement
LV: Left ventricle/left ventricular
MESA: Multiethnic Subclinical Atherosclerosis Study
MOLLI: Modified Look-Locker inversion recovery
NT-proBNP: n-terminal pro b-type natriuretic peptide
PSIR: Phase-sensitive inversion recovery
PWV: Pulse wave velocity
RA: Right atrium/right atrial
ROI: Regions of interest
RV: Right ventricle/right ventricular
SOP: Standard operating procedure
VENC: Velocity-encoded
VIBE: Volumetric interpolated breath-hold examination
